# 541. In Silico Modeling by Causal Inference for Identifying Biomarkers of Sepsis

**DOI:** 10.1093/ofid/ofac492.594

**Published:** 2022-12-15

**Authors:** Walisson Carvalho, Rita Silvério-Machado, Bráulio R G M Couto, Luis Enrique Zarate

**Affiliations:** Centro Universitário UNA, Belo Horizonte, Minas Gerais, Brazil; Universidade Federal de Minas Gerais - UFMG, Porto, Porto, Portugal; AMECI – Associação Mineira de Epidemiologia e Controle de Infecções, Belo Horizonte, Minas Gerais, Brazil; Universidade Católica de Minas Gerais - PUCMINAS, Belo Horizonte, Minas Gerais, Brazil

## Abstract

**Background:**

“Every 3 seconds, someone in the world dies of sepsis” (https://sepsistrust.org/about/). We used causal inference theory as a in silico method to identify biomarkers of sepsis. Causal Inference is a theory in Machine Learning that seeks for the root causes of an event.

**Methods:**

In the study it was used transcription profile data downloaded from http://www.ncbi.nlm.nih.gov/geo/query/acc.cgi?acc=GSE12624. This dataset has 70 samples, being 34 sepsis and 36 non-sepsis samples. Data set contains 8,519 attributes: 7,672 genes obtained after preprocessing of the mRNA expression profile data. The method applied in the dataset was a modification in the HEISA, a local learner of two stages algorithm (Figure 1). In the first stage HEISA identifies variables that compounds the set of parents, children, parents of parents and children of children of a target. During the second stage is calculated the causal effect using do-calculus method, of the selected variables of the first stage in the target. At end of the second stage, features with causal effect greater than 0.2 is selected. After selecting the features mRNA expression, it was applied two algorithms of classification, Random Forest and K-means, in order to evaluate the ability of the selected variables of identifying the occurrence of sepsis.

**Figure 1:**
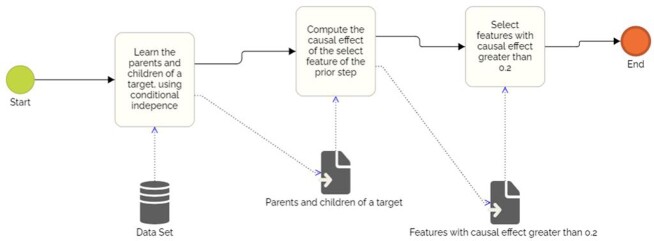
- Causal inference HEISA algorithm.

**Results:**

As shown in Table I, after applying the algorithm, it was select six mRNA expressions that better explains whether sepsis occurs, or not. Analyzing these genes it was possible to observe that three of them are known biomarkers, they are related to sepsis: NM_017526, NM_004649 and NM_006099. It is important to stress that the second one, Homo sapiens leptin receptor overlapping transcript (LEPROT) transcript variant 1 mRNA, is known to be inversely proportional to sepsis (its causal effect is negative). The other three biomarkers, NM_017526, NM_001274, and NM_001071 also are inversely proportional to sepsis. Their relationship with sepsis is still unknown. Regarding the classification task, using only those six mRNA expressions, the accuracy of the task of classification was 100%.

**Table I:**
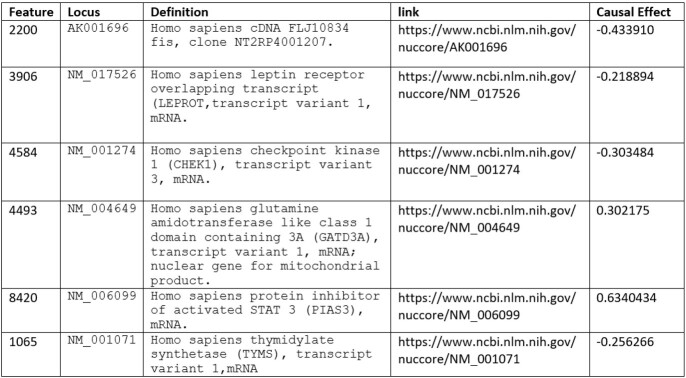
- Sepsis biomarkers candidates.

**Conclusion:**

In a big set of 7,672 genes, only six were returned as sepsis biomarkers candidates. This is a very promising in silico discovery, made by a novel mathematical method, the Causal Inference theory.

**Disclosures:**

**All Authors**: No reported disclosures.

